# Supplementary material for: Belt-type electrical muscle stimulation preserves muscle fiber size but does not improve muscle function in a rat model of cancer cachexia
Source: PLoS One. 2025 Nov 6;20(11):e0336391. doi: 10.1371/journal.pone.0336391 (PMC12591498; doi:10.1371/journal.pone.0336391)
Supplement: S1 Fig — (This would be included in the submission as a supplementary file). (PPTX) [file pone.0336391.s001.pptx]

## Slide 1
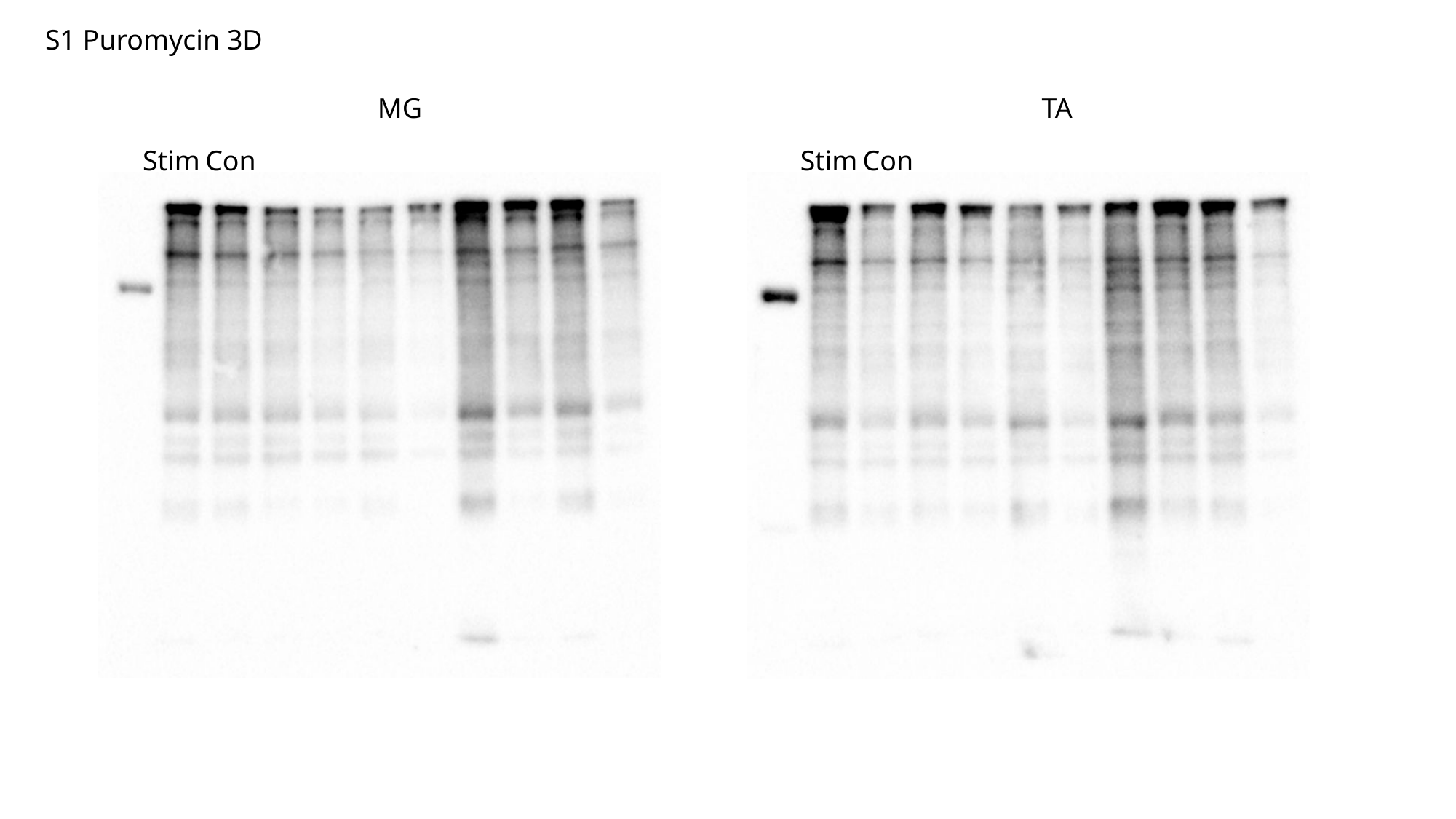

S1 Puromycin 3D
MG
TA
Stim
Con
Stim
Con

## Slide 2
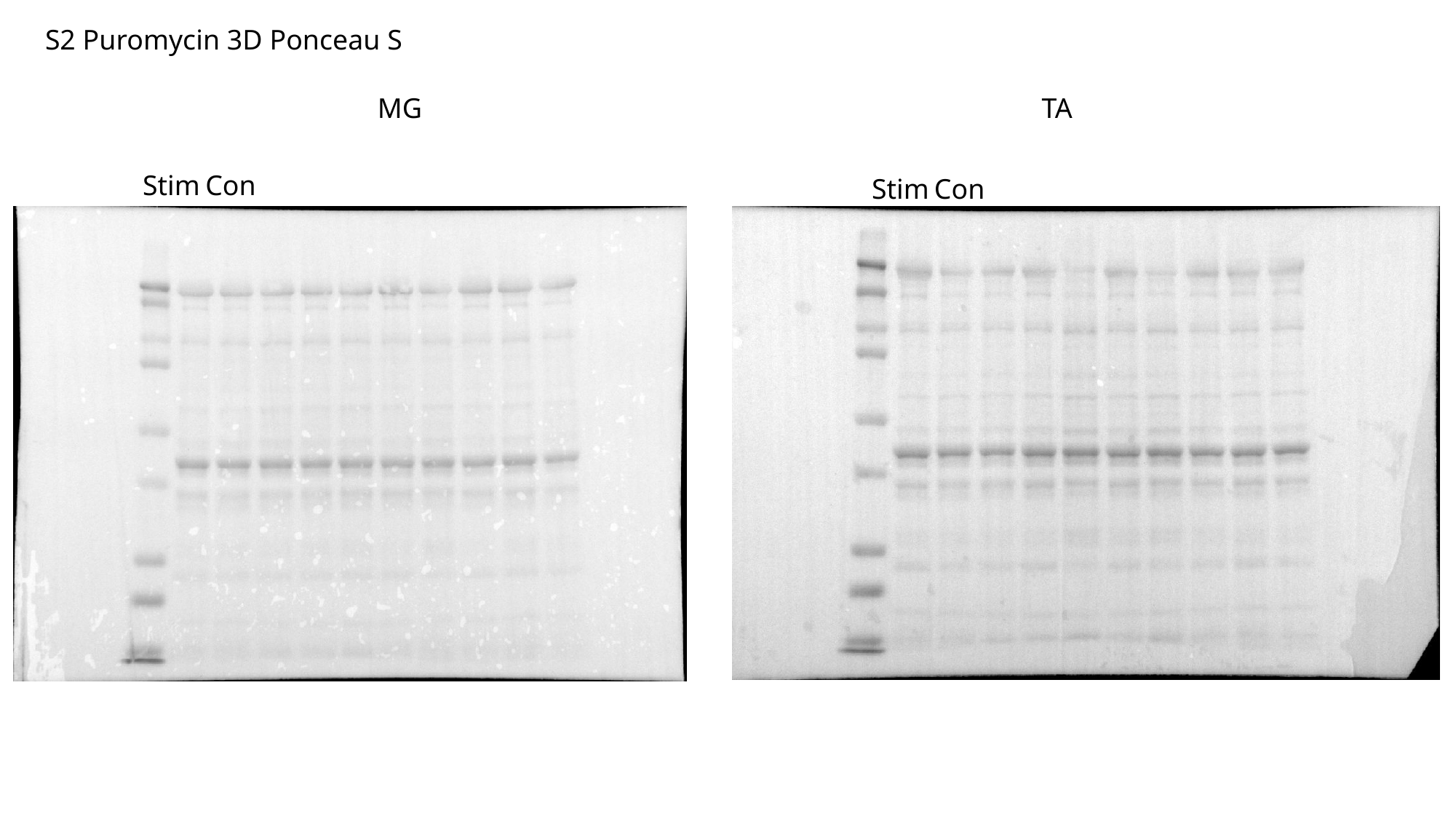

S2 Puromycin 3D Ponceau S
MG
TA
Stim
Con
Stim
Con

## Slide 3
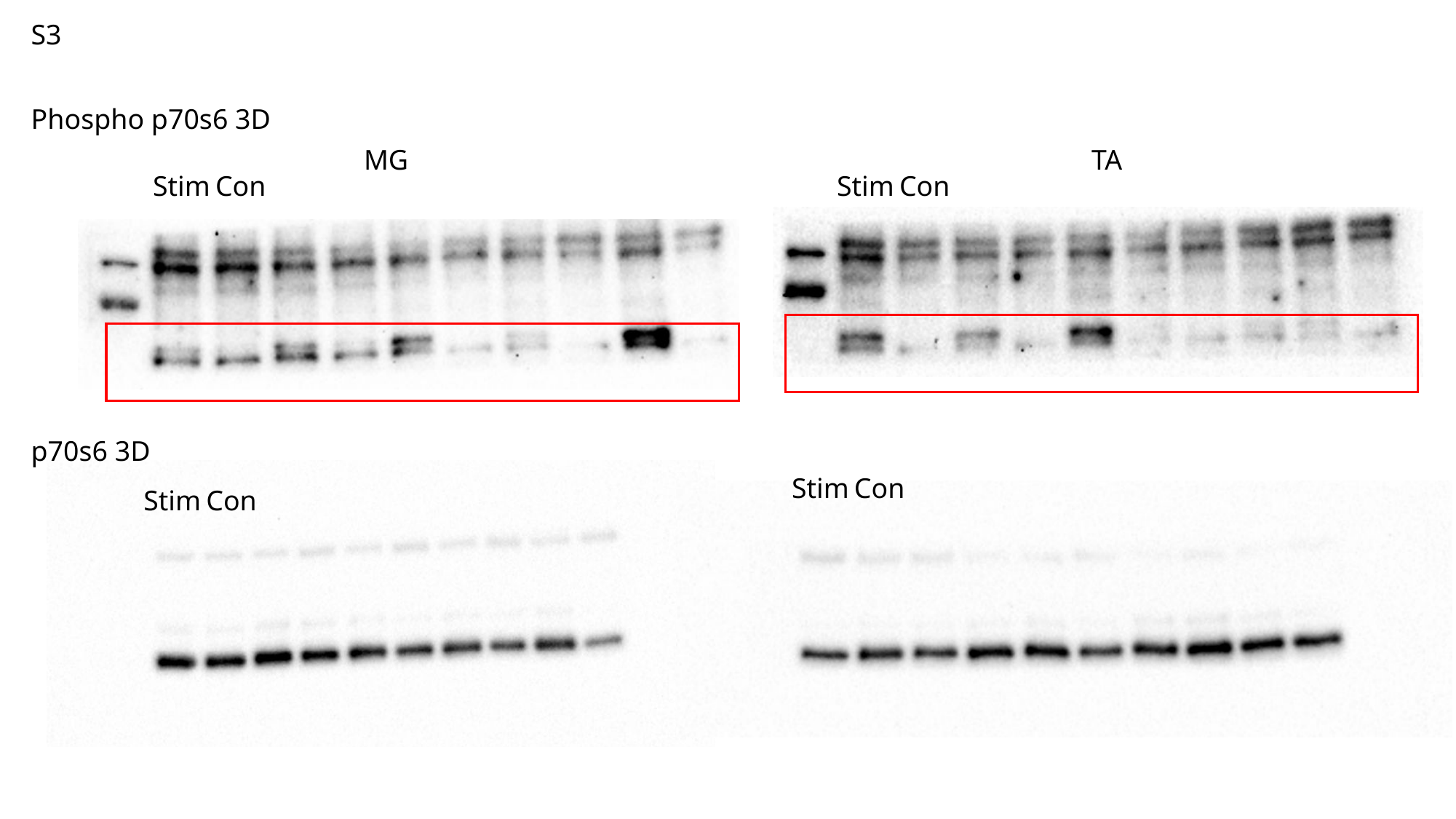

S3
Phospho p70s6 3D
MG
TA
Stim
Con
Stim
Con
p70s6 3D
Stim
Con
Stim
Con

## Slide 4
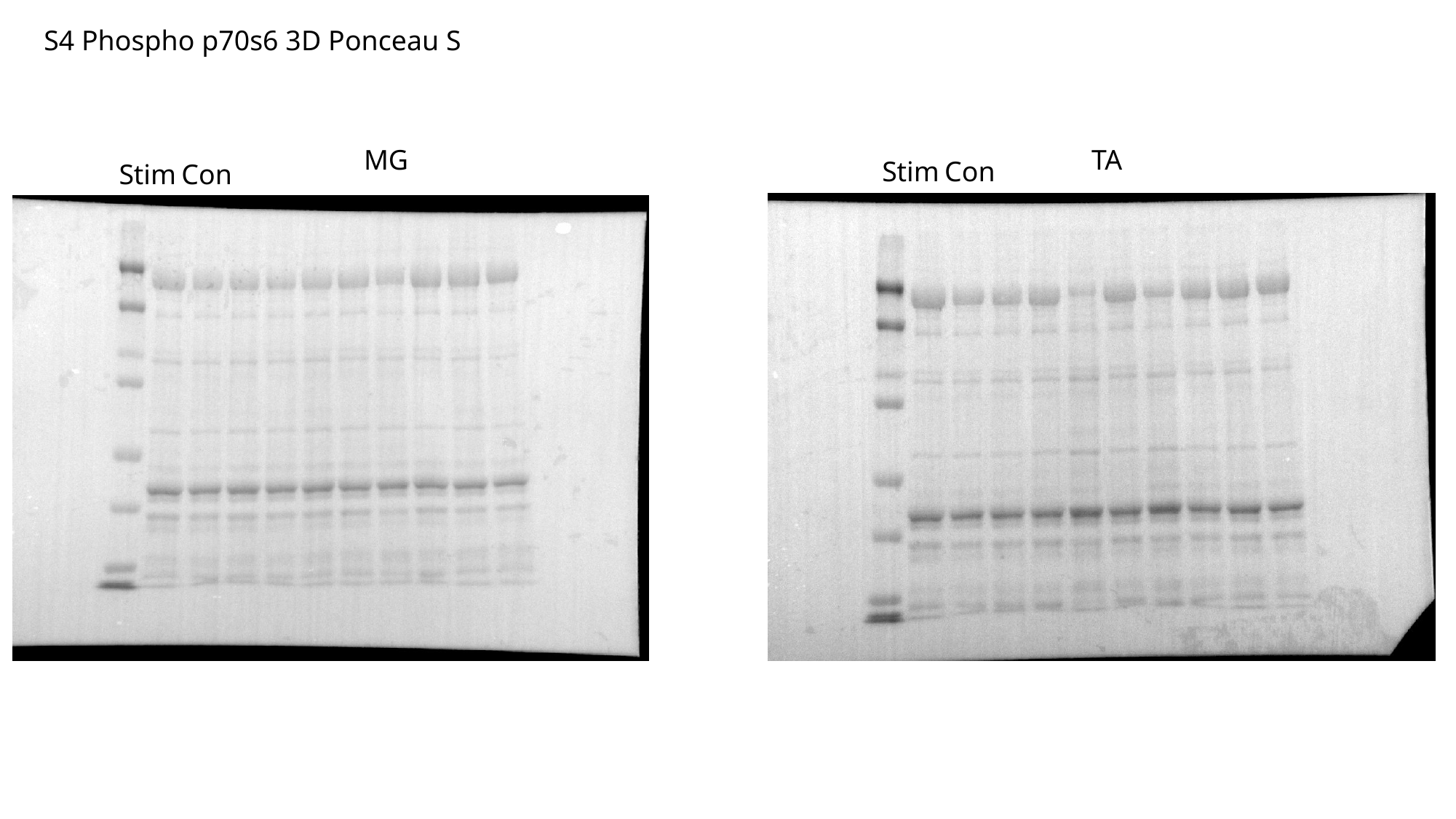

S4 Phospho p70s6 3D Ponceau S
MG
TA
Stim
Con
Stim
Con

## Slide 5
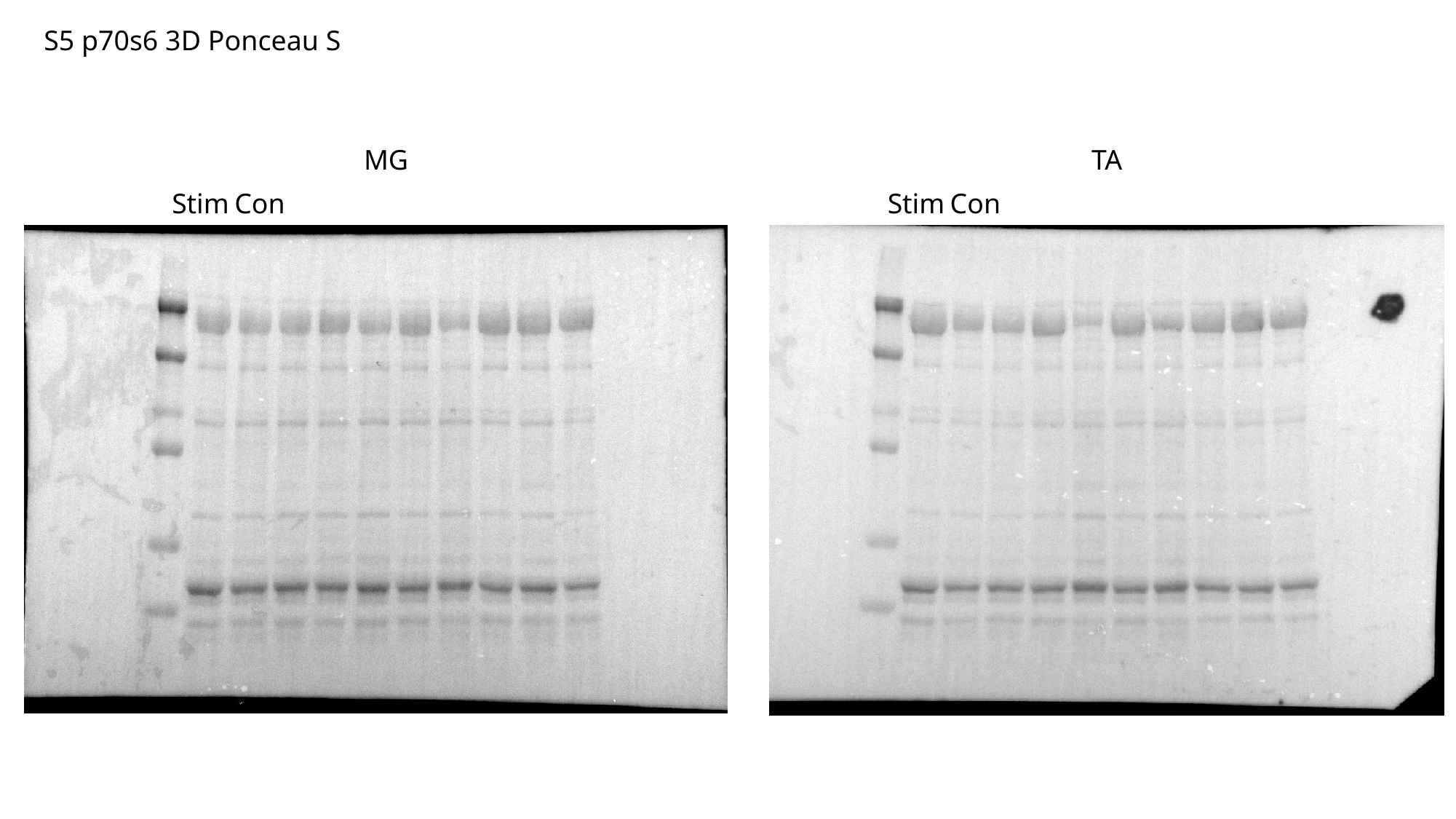

S5 p70s6 3D Ponceau S
MG
TA
Stim
Con
Stim
Con

## Slide 6
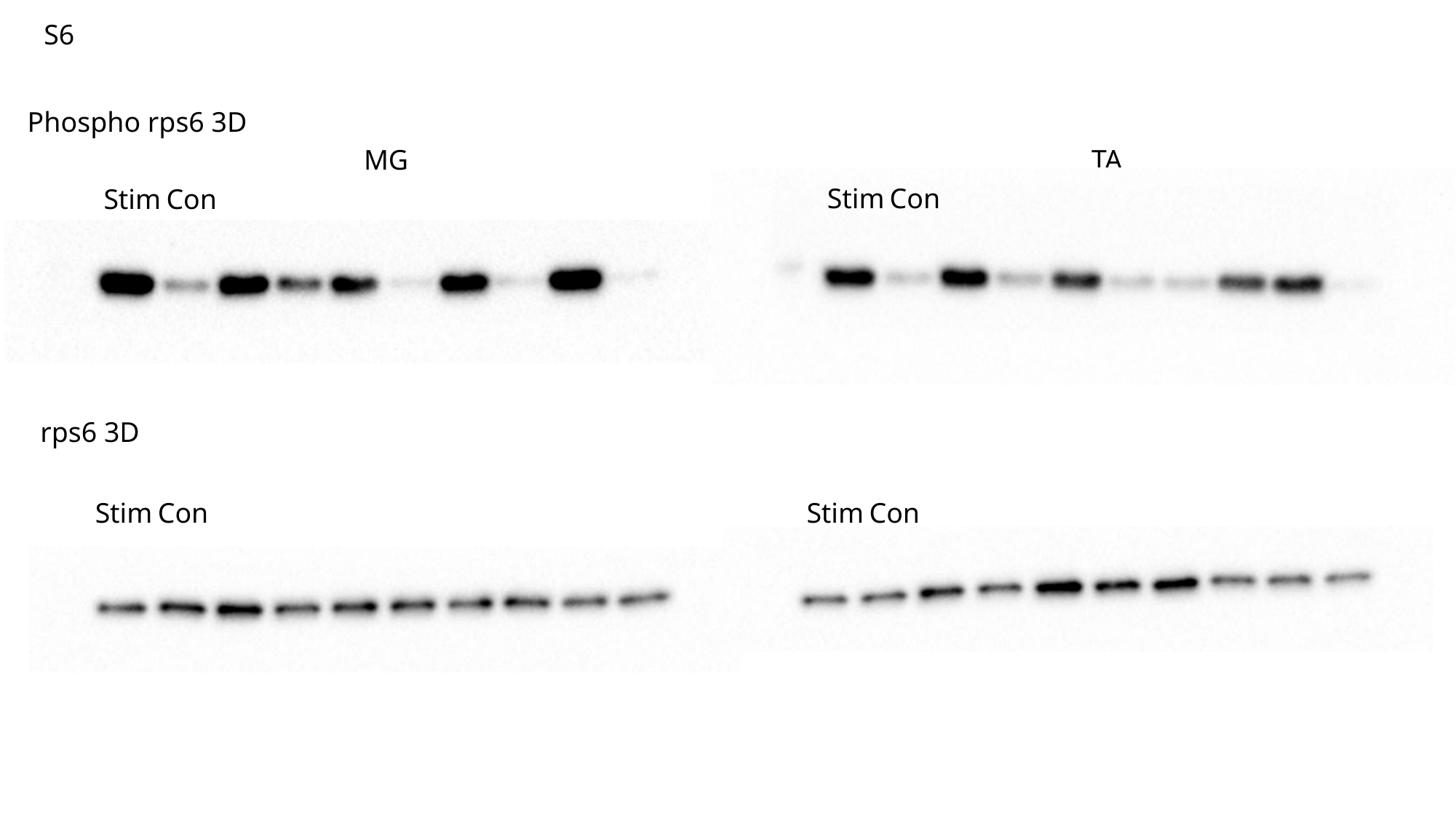

S6
Phospho rps6 3D
MG
TA
Stim
Con
Stim
Con
rps6 3D
Stim
Con
Stim
Con

## Slide 7
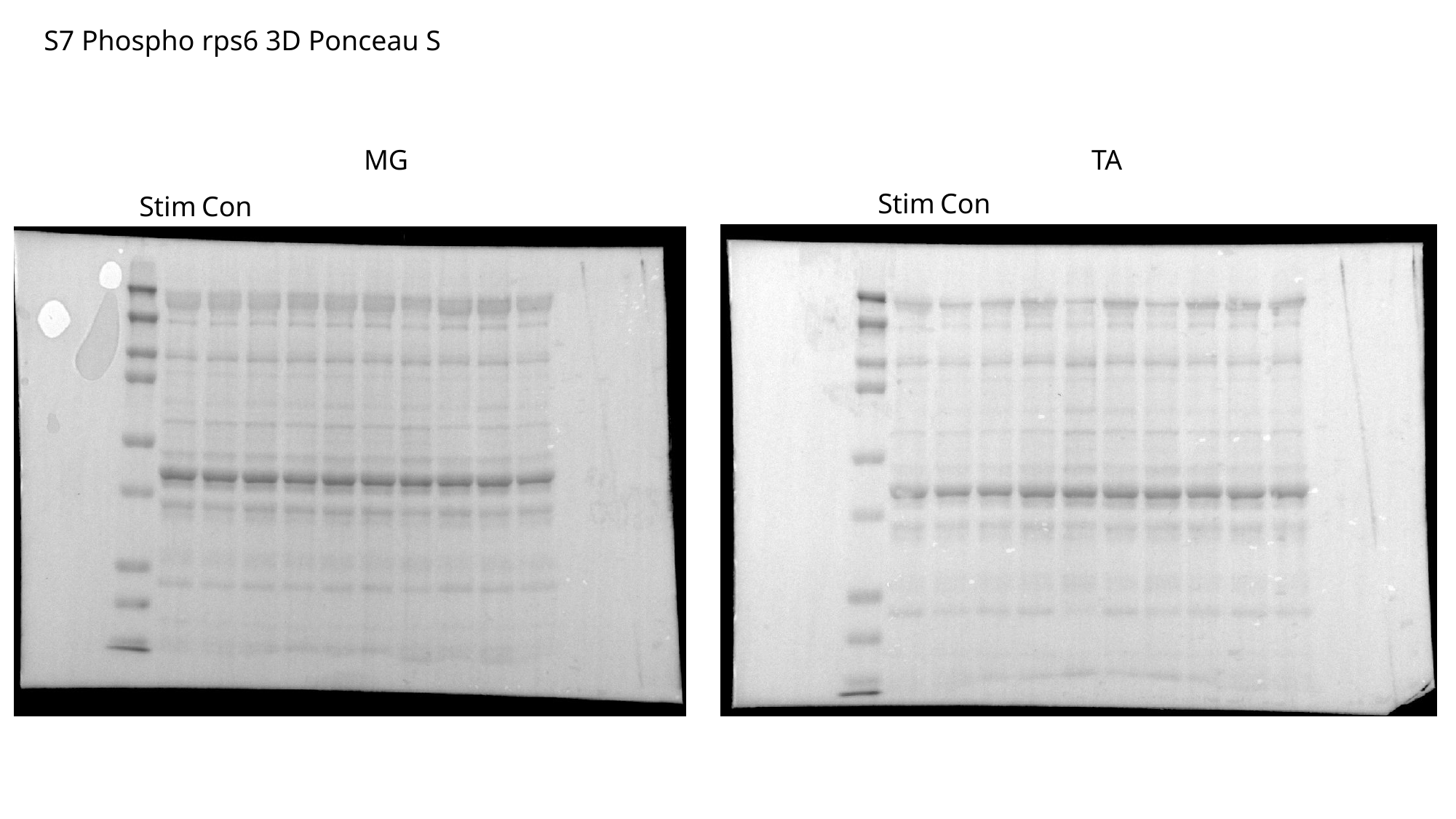

S7 Phospho rps6 3D Ponceau S
MG
TA
Stim
Con
Stim
Con

## Slide 8
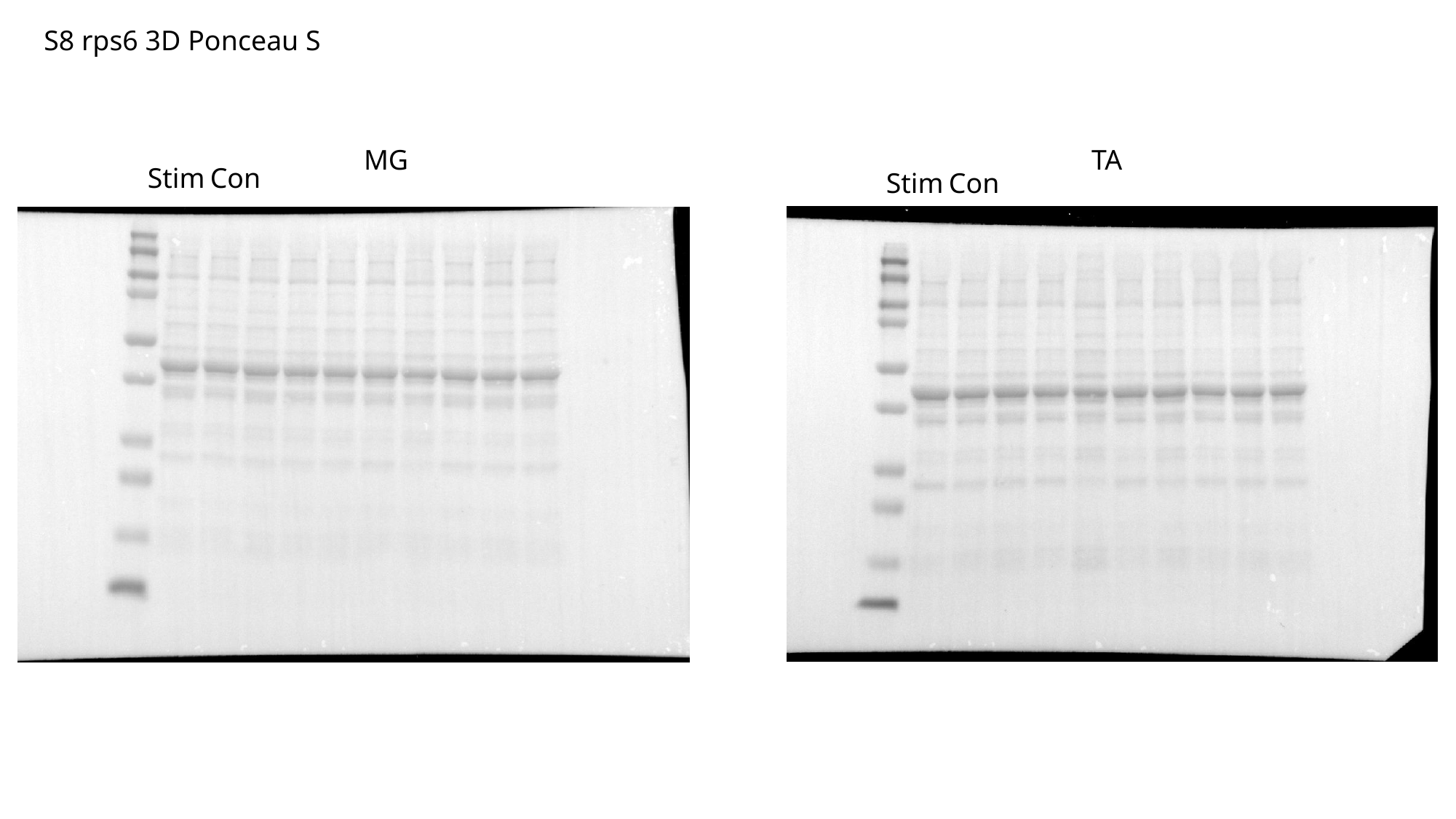

S8 rps6 3D Ponceau S
MG
TA
Stim
Con
Stim
Con

## Slide 9
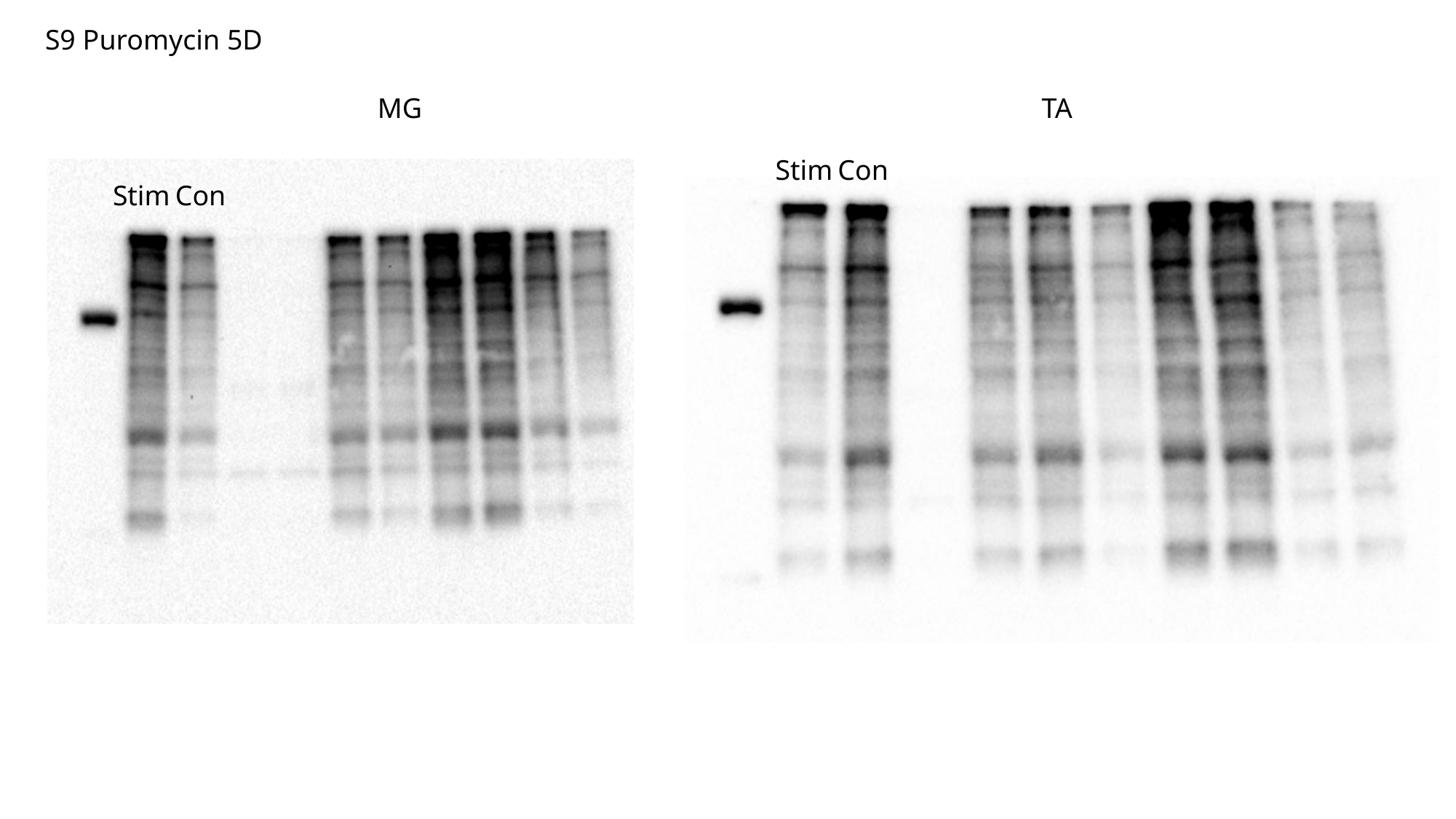

S9 Puromycin 5D
MG
TA
Stim
Con
Stim
Con

## Slide 10
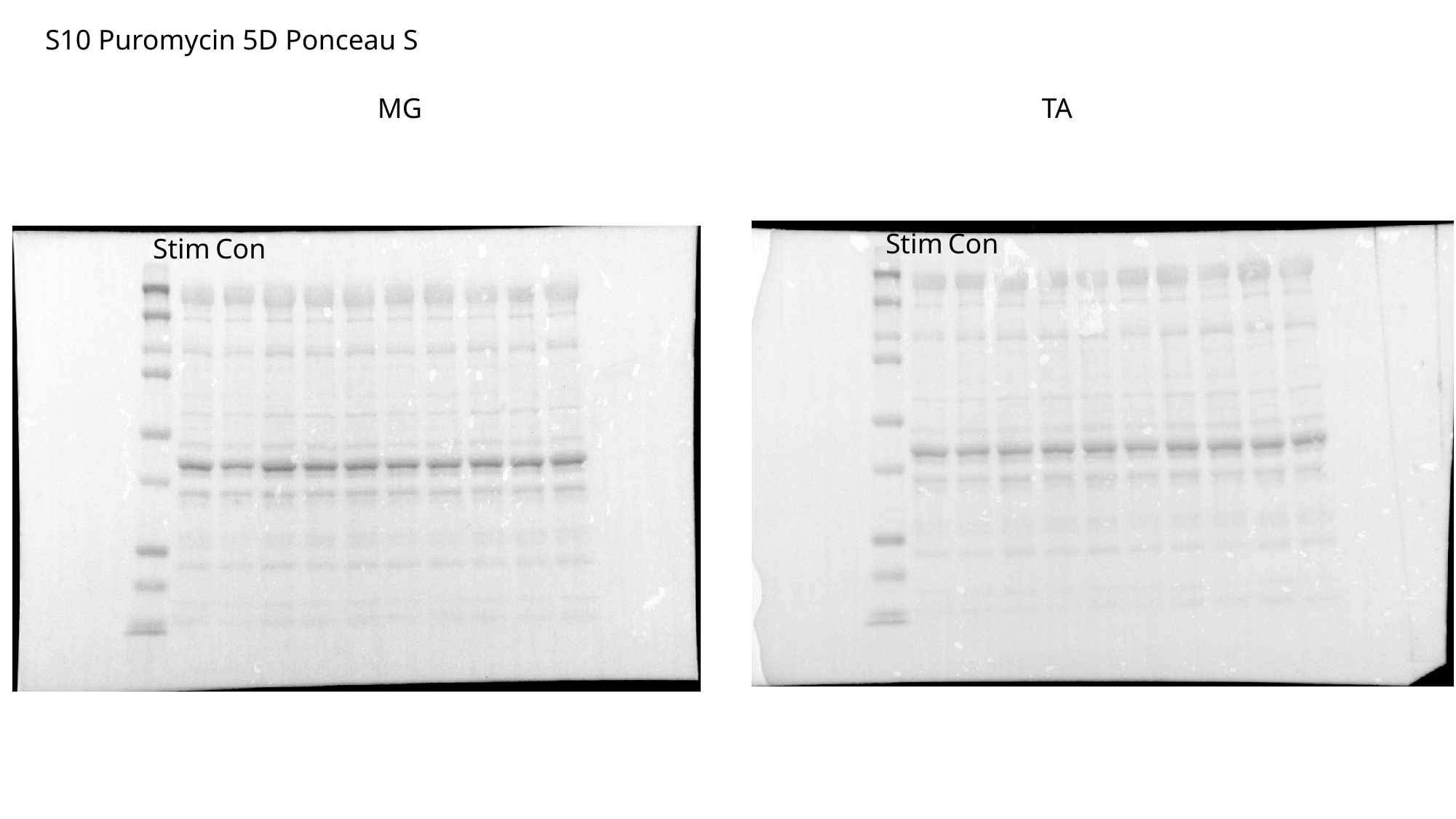

S10 Puromycin 5D Ponceau S
MG
TA
Stim
Con
Stim
Con

## Slide 11
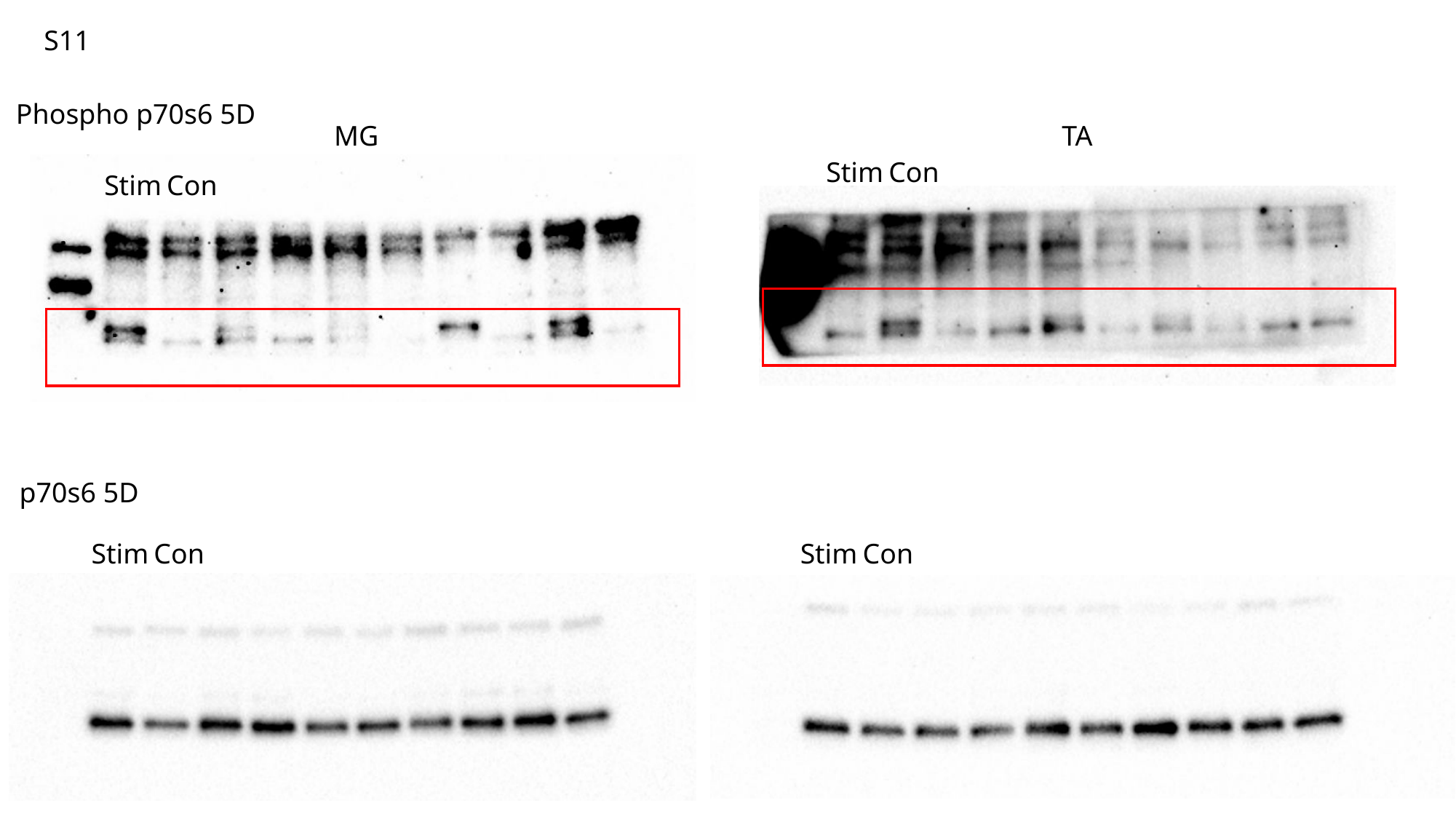

S11
Phospho p70s6 5D
MG
TA
Stim
Con
Stim
Con
p70s6 5D
Stim
Con
Stim
Con

## Slide 12
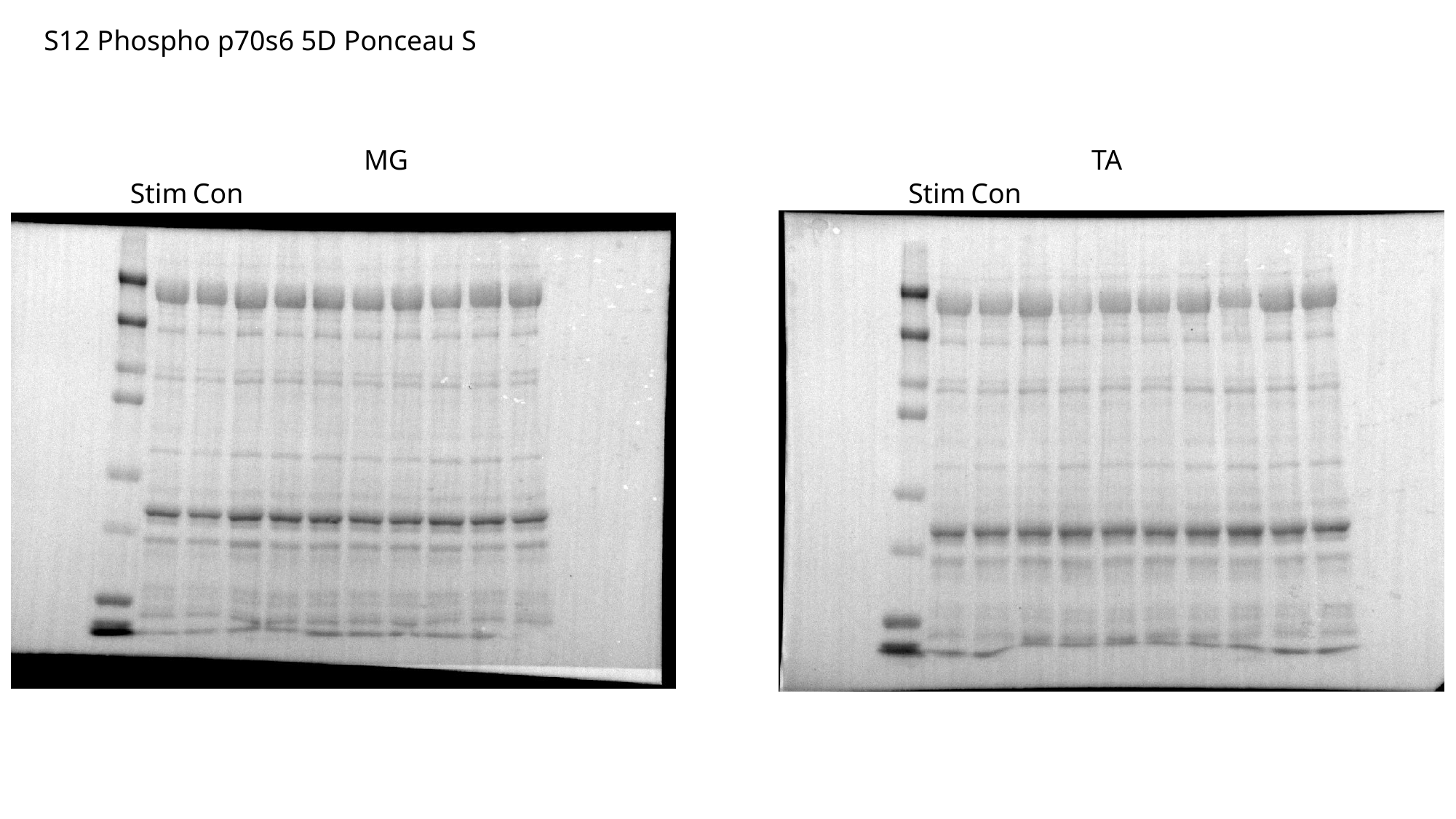

S12 Phospho p70s6 5D Ponceau S
MG
TA
Stim
Con
Stim
Con

## Slide 13
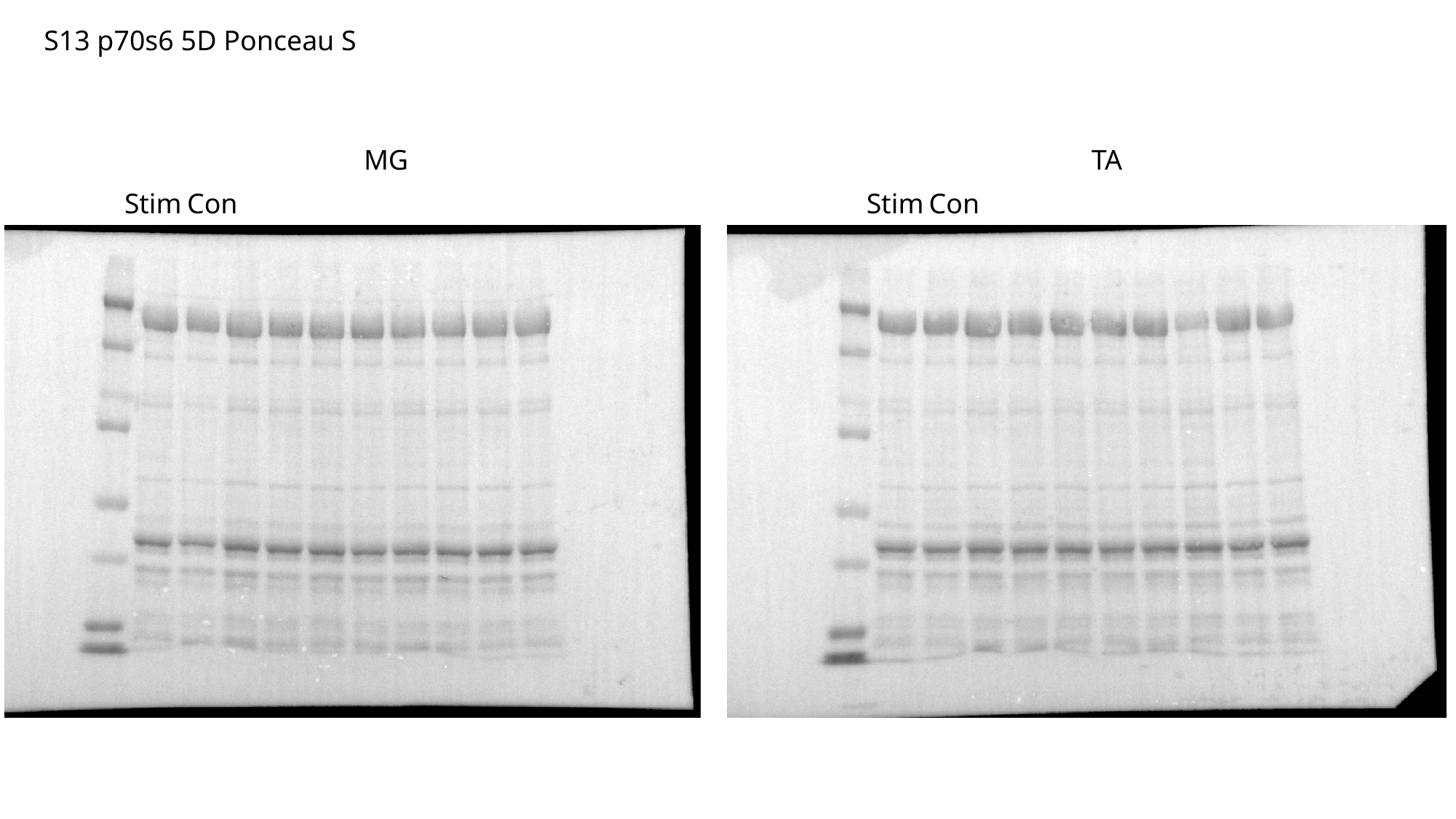

S13 p70s6 5D Ponceau S
MG
TA
Stim
Con
Stim
Con

## Slide 14
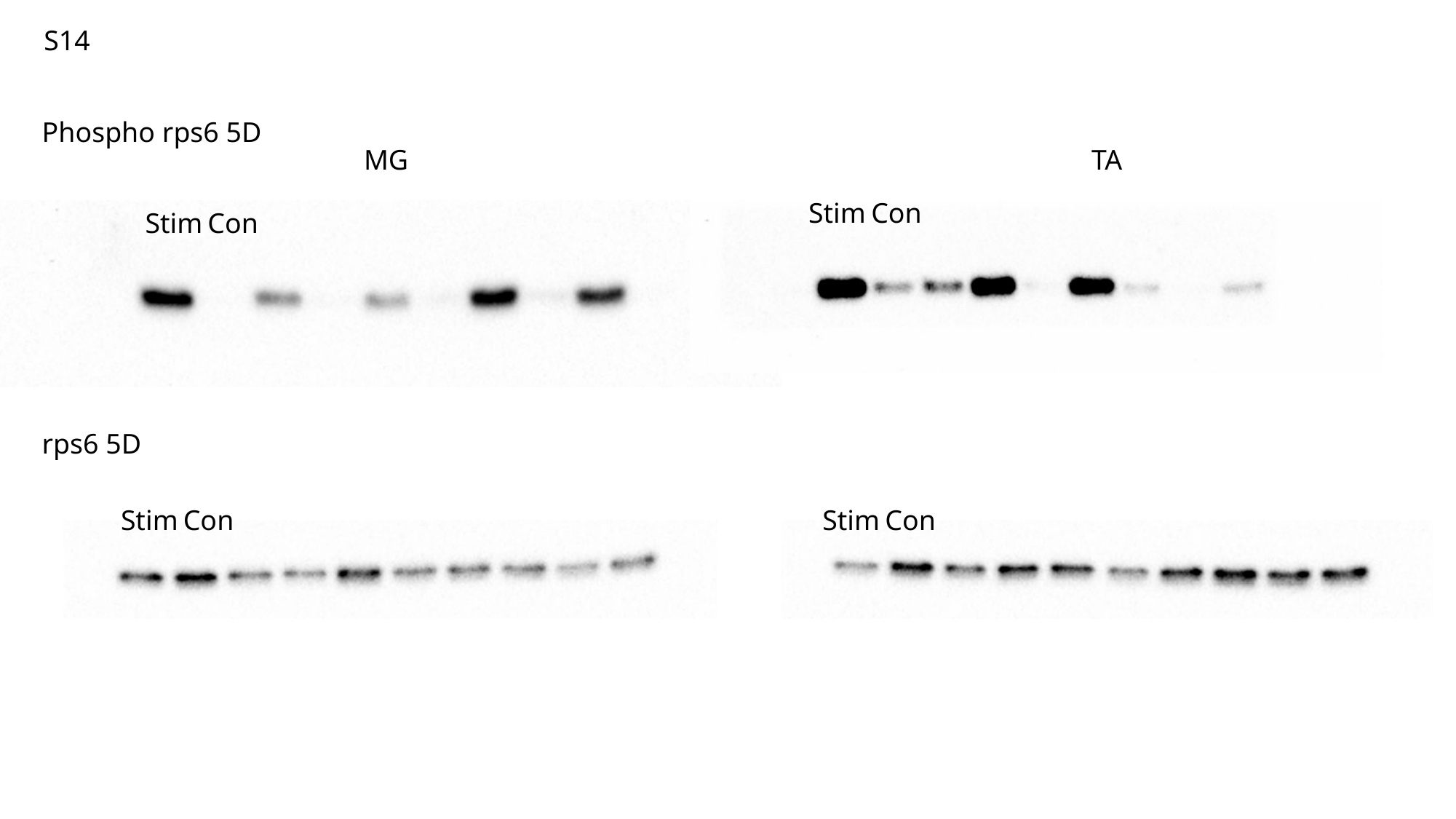

S14
Phospho rps6 5D
MG
TA
Stim
Con
Stim
Con
rps6 5D
Stim
Con
Stim
Con

## Slide 15
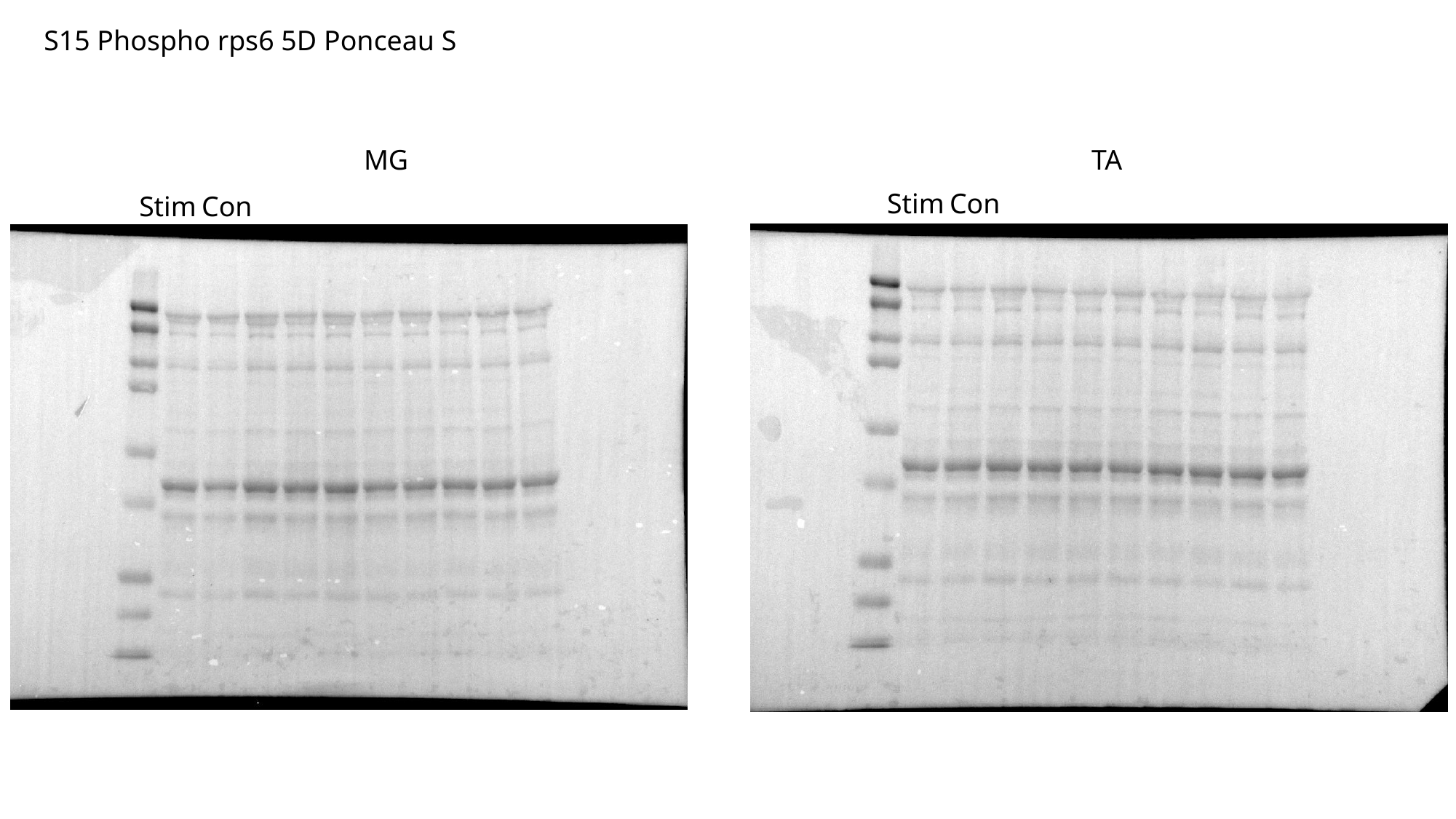

S15 Phospho rps6 5D Ponceau S
MG
TA
Stim
Con
Stim
Con

## Slide 16
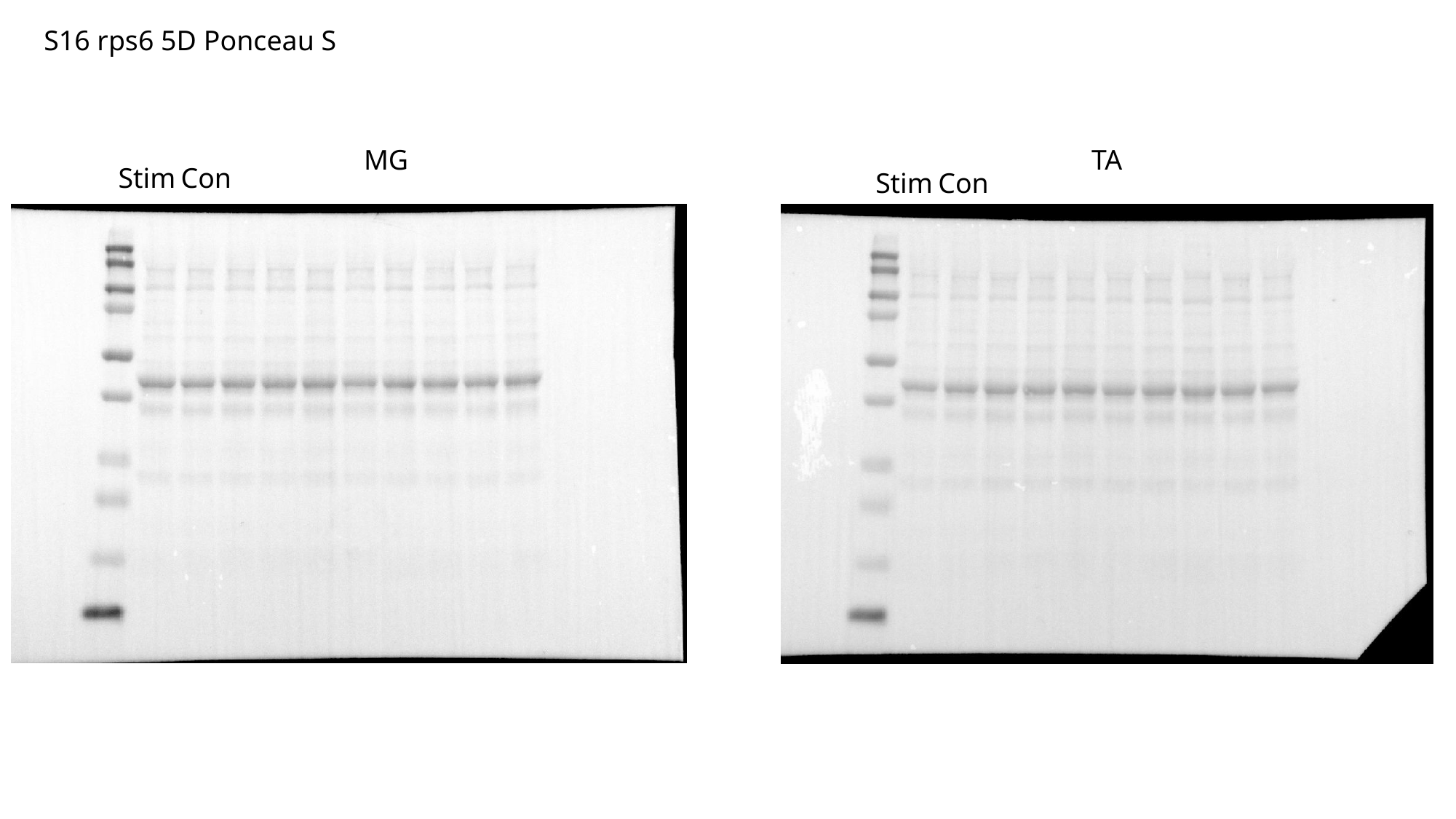

S16 rps6 5D Ponceau S
MG
TA
Stim
Con
Stim
Con
